# Cognitive assessment in patients with multiple sclerosis: A Spanish consensus

**DOI:** 10.3389/fresc.2022.1006699

**Published:** 2022-12-20

**Authors:** Yolanda Higueras, Mónica Borges, Isabel Jiménez-Martín, Cristina Conde, Ana Aparicio-Mingueza, Esther Sierra-Martínez, Jordi Gich-Fulla, Marta Balaguer-Marmaña, Anna Gil-Sánchez, Elisenda Anglada, Ana Jover, María Yaiza Pérez-Martín, María Jesús Arévalo, Carlos Arrabal-Gómez, Judith Jiménez-Veiga, Genny Lubrini, Ana Molano, Fabiola García-Vaz

**Affiliations:** ^1^ Department of Neurology, Instituto de Investigación Sanitaria Gregorio Marañón, Hospital General Universitario Gregorio Marañón, Madrid, Spain; ^2^ Department of Cognitive Processes and Speech Therapy, School of Psychology, Experimental Psychology, Complutense University, Madrid, Spain; ^3^ Department of Neurology, Virgen Macarena Hospital, Seville, Spain; ^4^ Department of Neurology, Health Research Institute Foundation of Santiago de Compostela (FIDIS), University Clinical Hospital from Santiago de Compostela, Santiago de Compostela, Spain; ^5^ Neurology Service, Maimonides Institute for Research in Biomedicine of Cordoba, (IMIBIC), Reina Sofía University Hospital, Cordoba, Spain; ^6^ Department of Psychology, ACOAD Association, Pamplona, Spain; ^7^ Department of Neurorehabilitation, Hospital San Juan de Dios, Zaragoza, Spain; ^8^ Fundación Neurópolis, Zaragoza, Spain; ^9^ Department of Neurology, Dr. Josep Trueta University Hospital, Girona, Spain; ^10^ Department of Neurology, Consorci Sanitari Integral Moisés Broggi Hospital, Sant Joan Despí, Spain; ^11^ Department of Neuroimmunology, Biomedicine Research Institute of Lleida, Lleida, Spain; ^12^ Department of Neurology-Neuroimmunology, Multiple Sclerosis Centre of Catalonia, Hospital Universitari Vall d'Hebron, Barcelona, Spain; ^13^ Department of Neurosurgery, Hospital Universitario de Canarias, Santa Cruz de Tenerife, Spain; ^14^ Department of Psychobiology and Methodology of Behavioral Sciences, University of Málaga, Málaga, Spain; ^15^ Department of Neurology, Hospital Clínico Universitario Virgen de la Arrixaca, Murcia, Spain; ^16^ Department of Neurology, Hospital de Cruces, Bilbao, Spain; ^17^ Multiple Sclerosis Fundation, Madrid, Spain

**Keywords:** neuropsychologial assessment, multiple sclerosis, follow-up, cognition, consensus, cognitive assessment, cognitive screening

## Introduction

Multiple Sclerosis (MS) is an inflammatory, degenerative, and autoimmune disease of the central nervous system causing myelin loss, axonal degeneration, and brain atrophy. Although MS is often diagnosed at second or third decade of life, it can develop at any age (1). The symptomatology is heterogeneous and includes cognitive impairment (CI) occurring in up to 70% of all patients with MS (2). CI can occur in the early stages of disease even in the absence of neurological deficits (3, 4). Deficits in information processing speed, attention, verbal and visual learning, verbal and visuospatial functions, and executive functions are thought to be the most affected cognitive functions in MS (2). CI occurs in all MS disease phenotypes although its prevalence tends to be higher in the progressive forms of the disease as compared to the relapsing forms (5, 6).

Several studies have shown that CI produces a negative impact on the patients' quality of life, work, and social functioning (7, 8). Early detection of CI has become an important aspect to be considered for an adequate follow-up, to optimize social adaptation and to implement specific cognitive rehabilitation strategies (9). This is why it is of importance in routine clinical practice aiming to perform both type of assessments, i.e., screening tools and comprehensive neuropsychological test batteries assessing both cognitive and emotional functioning and quality of life.

The Neuropsicored-EM group includes 18 neuropsychologists from different healthcare centers all over Spain. The goal of the present work is twofold. On the one hand, to propose a holistic approach to CI in MS. On the other, to provide recommendations for the assessment and follow-up of cognitive function in adult MS subjects. The use of a common evaluation protocol across Spanish centers engaged in MS management would facilitate the development of multicentric studies and promote network research in the Spanish National Health System.

## Cognitive impairment in multiple sclerosis

Slowed information processing speed is the hallmark and the first cognitive domain to be altered in MS patients, detectable even in the asymptomatic phase of the disease (4). Information processing speed deficits are considered as an early diagnostic marker (10) and a useful indicator of both disease progression (11) and functional impairment (12).

Learning and memory impairments are also common among persons with MS (2). However, research on the nature of MS-related memory problems is mixed. Two hypotheses have been proposed. While some authors have argued for deficient retrieval processes as the core memory deficit (13) others have identified poor initial learning as the core deficit (14, 15), which would partially explain poor delayed retrieval (16).

Deficits in cognitive flexibility, inhibition, and abstraction occur in 20%–80% of MS patients (17, 18). Executive functions deficit in MS have been only partially studied (19), as most broadly utilized batteries do not include tests assessing executive functions (e.g., the Brief International Cognitive Assessment in Multiple Sclerosis, BICAMS) or focus on executive functions subdomain, usually verbal fluency (e.g., the Brief Repeatable Battery Neuropsychology BRB-N).

Visuospatial and visuoperceptual functions have been shown to be affected regardless of other visual deficits (20). However, most batteries usually used in MS do not include tests assessing these cognitive domains.

Mental health comorbidities in MS patients (i.e., depression, anxiety) has been proved to be related with an increased risk of suicide, poorer quality of life, cognitive deficits, problems at work and poor adherence to a disease-modifying treatment (21, 22). Fatigue is one of the most common symptoms of MS, occurring in 75%–95% of patients and at all stages of the disease. It is reported by patients as one of the most disabling symptoms, interfering with work functioning (23–25) and it is strongly related with the emotional state (25–27).

Cognitive reserve is considered a key aspect to take into account during neuropsychological evaluations aimed at detecting cognitive impairment. It should also be considered when monitoring the progression of cognitive impairment (28).

## Proposal for evaluation

This proposal is based on international experts' opinions and the best available evidence about cognitive assessment in MS (29, 30). Moreover, the present recommendations aim to overcome some limitations of previously proposed assessment protocols, providing more executive measurements and MS specific patient rating outcomes for quality of life, fatigue impact and emotional status for a broader assessment.

A trained neuropsychologist should administer the basic assessment protocol and interpret the results. This protocol should be administered at baseline (first referral of the patient) to allow future comparisons of patient's cognitive profile during follow-up (11, 29–31). When a trained neuropsychologist is not available, administration of the Symbol Digit Modalities Test as a rapid screening test to broadly detect cognitive impairment is suggested.

The basic evaluation protocol includes the following tests:

**Selective reminding test (SRT)**: this test assesses learning ability and verbal long-term retention. It distinguishes between short-term and long-term memory, and learning or retrieving information difficulties.

**Spatial recall test (10/36 SPART)**: this test assesses learning capacity and long-term visuospatial retention. SPART Immediate Recall score is the product of the total number of correct responses (i.e., number of correct checkers) for the three learning trials. SPART Delayed Recall score is the total number of correct responses (i.e., number of correct checkers) in the delay condition.

**Symbol digit modalities test (SDMT)**: this test is a measure of sustained attention, processing speed, visual scanning, and motor speed. The number of correct substitutions within 90 s is recorded. In the written version of the test the subject fills in the numbers that correspond to the symbols. In an oral version, the examiner records the numbers spoken by the subject.

**Paced auditory serial addition task (PASAT)**: this test evaluates sustained and divided attention, information processing speed, working memory and calculation abilities. This test can be given at different rates of presentation ranging from a slow rate of one number every 2.4 s to the fastest rate of one number every 1.2 s. The total score is the number of correct responses.

**Cued verbal fluency task** (based on the Word List Generation): the original test evaluates semantic verbal fluency through categorial evocation. We also propose to assess phonetic verbal fluency by two additional trials in which participants have to produce as many words as possible starting with a given letter (P) and as many words as possible without a given letter (word without E). The total number of correct words evoked in each trial is scored, as well as intrusions and perseverations.

**Trail making test (TMT)**: this test assesses cognitive flexibility, visuomotor processing speed, visual search, and working memory. Results for both TMT A and B are reported as the number of seconds required to complete the task.

**Working memory index (WAIS IV)**: this test provides a measure of a person's ability to attend to information presented verbally, manipulate that information in short-term immediate memory, and then formulate a response. It consists of 3 subtests: Digit Span Forward (individual tries to repeat digits forward); Digit Span Backward (individual tries to repeat digits backward), and Digit Span Sequencing (individual tries to repeat digits in ascending order).

**Hospital anxiety and depression scale (HADS)**: it is a self-administered questionnaire designed to assess anxiety and depression symptoms in medical patients, with emphasis on reducing the impact of physical illness on the total score. The HADS produces two scales, one for anxiety (HADS–A) and one for depression (HADS–D), differentiating the two states. The items are scored on a 4-point Likert frequency scale (0–3), with a total score ranging from 0 to 21 on each subscale, where a higher score is indicative of greater symptom severity.

**Multiple sclerosis quality of life-54 (MSQoL-54)**: it is a quality-of-life questionnaire containing items about physical function, bodily pain, general health, vitality, social function, emotional state and mental health, rest, health concern, function sexual activity, cognitive activity and quality of life in general. Scores goes from 0 to 100, the higher the better.

**Multiple sclerosis fatigue severity scale (MSFSS)**: it is a scale which measures both the severity of fatigue and its effect on the person's activities and lifestyle. Rates the impact of fatigue in physical, cognitive and social activity, from 0 (not at all) to 84 (maximum interference).

When a more comprehensive neuropsychological evaluation is necessary, the basic evaluation protocol should be complemented by the administration of the following tests:

**Prefrontal symptom inventory (PSI)**: it is a self-administered questionnaire designed to assess cognitive, emotional and behavioral alterations related to prefrontal dysfunction.

**Environmental status scale (EES)**: it is a scale used to evaluate the following seven parameters: actual work status, financial and economic status, personal residence or home, required personal assistance, transportation, community services, and social activity.

**Benton's line orientation judgment (JOLB)**: this measure is a motor-free determinant of visual-spatial skill.

**Nine hole pegboard test (9-HPT)**: it is used for the assessment of manual dexterity. The total time to complete the task is recorded. Two consecutive trials with the dominant hand are followed by two consecutive trials with the non-dominant hand.

**The Rey–Osterrieth complex figure (ROCF)**: the Boston Qualitative Scoring System for the ROCF includes five scores (Planning, Fragmentation, Neatness, Perseveration, and Organization) developed to measure the executive aspects of ROCF productions (Stern et al., 1994).

**Stoop color and word test (STROOP)**: it is used to evaluate speed of visual search, working memory, cognitive flexibility, and conflict monitoring. The number of correct responses within 45 s in each of the three trials of the test are recorded, as well as the derived Interference Score.

**Beck depression inventory (BDI-II)**: it is a self-administered questionnaire used to evaluate the presence of depressive symptoms. The total value of the BDI can range from 0 to 63 points. Higher total scores indicate more severe depressive symptoms.

**State-trait anxiety inventory (STAI)**: it is a self-administered questionnaire that measures state and trait anxiety.

**Cognitive reserve questionnaire (CRC)**: it is an instrument for measuring the cognitive reserve (Rami et al., 2011).

**Vocabulary subtest (WAIS-IV)**: this subtest assesses the patient's understanding of words and reflects language development, expressive language skills, cultural and educational experiences, ability to use words appropriately, and retrieval of information from long-term memory.

During the administration of the above-mentioned assessment protocols, professionals should take into account the following recommendations:

1. In order to avoid test–retest learning effects, parallel forms of the same test should be administered. Follow-up evaluations are not recommended before 6 months of the basal one.
2. Regarding the recommended periodicity for the follow-up, evaluations sessions should be spaced according to the clinical characteristics of the patient. If they present a mild to moderate cognitive impairment at baseline, annual reassessments are encouraged to determine any possible progression or, on the contrary, the stabilization of the neuropsychological situation (9).
3. To appropriately interpret the results of the evaluations, both the information gathered during the initial clinical interview, as well as the assessment regarding behavioral pattern and the patients' performance on tests, should be considered.
4. When conducting the neuropsychological evaluations, patients’ test scores should be compared to appropriate normative data to maximize diagnostic and descriptive accuracy. We recommend using the normative data by Sepulcre et al. (32) for the SRT and the SPART; the normative data by Peña Casanova et al. (33) for the SDMT, Digit Span Forward and Backward, TMT, JOLB, Stroop, and the verbal fluency test; the normative data by Fréderique Vallar et al. (34) for the WAIS-IV Vocabulary subtest and the Working Memory Index; the normative data by Rao et al. (7) for the PASAT; and the normative data available from each of manuals corresponding to the remaining tests.
5. Following the completion of a neuropsychological assessment, a neuropsychological report should be generated to communicate the findings of the evaluation. The report should at least incorporate the following sections: reason for referral, interview and observations, neuropsychological test results, conclusions and treatment recommendations. Neuropsychological reports should clearly summarize the results in a manner that is both useful to the patient and to the referral source (patient and his family, neurologist, lawyers).

## Conclusions

In the past few decades, the number of studies including neuropsychological data within the evaluation protocols for patients with MS have significantly increased. Moreover, it has been noted the importance of considering emotional, quality of life and fatigue measures as potential indicators of disease progression (35). In daily clinical practice, is key to optimize the evaluation process finding the right balance between the amount of information obtained and the effort and time invested.

With that in mind, the aim of this consensus paper is to describe the state of the art about cognitive assessment of patients with MS with the purpose of providing recommendations both for clinical purposes (diagnosis, evolution, effect of treatments) and the development of online research in MS. Considering that in Spain there are no formal guidelines published on the assessment of cognitive function in MS, we believe that the present work, which follows the proposals of other international reference groups (29, 30), would contribute to the development of an national consensus that could be used in both clinical and research fields to push further both investigation and personalized medicine for this patients.

However, there are still several goals to accomplish to establish a standard assessment procedure in MS. Evaluation is the first step, but there are other consensus challenges ahead that should be addressed to improve the MS research and care network.
